# Examining Grey Matter Structural Abnormalities in Young People Exposed to Childhood Maltreatment and Peer Victimisation

**DOI:** 10.1192/bjo.2022.210

**Published:** 2022-06-20

**Authors:** Lena Lim

**Affiliations:** SICS, ASTAR, Singapore, Singapore. IoPPN, King's College London, London, United Kingdom

## Abstract

**Aims:**

Early-life interpersonal stress, particularly childhood maltreatment (CM), is associated with neurobiological abnormalities. However, few studies have investigated the neural effects of peer victimisation (PV). This study examines the common and specific associations between CM, PV and brain structural alterations in healthy youths.

**Methods:**

Grey matter volume (GMV) and cortical thickness (CT) data were collected from 105 age-and gender-matched healthy youths (34 CM, 35 PV and 36 controls). Region-of-interest (ROI) and whole-brain analyses were conducted.

**Results:**

For the ROI, the CM group had smaller GMV than controls in left IFG, bilateral anterior insula, postcentral and lingual regions, which were associated with higher emotional abuse, along with smaller insular GMV than the PV group. The PV group had smaller left lingual GMV than controls, which was positively associated with age of bully onset. At the whole-brain level, both CM and PV groups had smaller GMV than controls in a cluster comprising left post/pre-central, inferior frontal, insula, superior parietal and supramarginal gyri. The PV group alone had increased CT in a cluster comprising left superior frontal, anterior cingulate and medial orbitofrontal gyri, which was related to greater cyberbullying.

**Conclusion:**

Early-life interpersonal stress from carers and peers is associated with common structural alterations of the inferior frontal-limbic, sensory and lingual regions involved in cognitive control, emotion and sensory processing. The findings of a CM-specific reduced anterior insular GMV and a PV-specific increased CT in the left medial prefrontal cluster is intriguing and underscores the unique negative effects of CM and PV, particularly cyberbullying.

